# The role of elastic intramedullary nailing with or without Kirschner wire fixation in pediatric radial neck fractures: a retrospective cohort study

**DOI:** 10.3389/fped.2026.1781399

**Published:** 2026-03-25

**Authors:** Zonghui Dai, Jian Xu, Mingjing Li, Jiang Xiang, Chunquan Zhu, Fucheng Ouyang, Sen Tang, Jiawen Yu, Fan Li

**Affiliations:** Department of Pediatric Orthopedics, Wuhan Fourth Hospital, Wuhan, Hubei, China

**Keywords:** elastic intramedullary nailing, fracture union, functional outcome, Kirschner wire fixation, pediatric radial neck fracture

## Abstract

**Objective:**

To compare the clinical and functional outcomes of elastic intramedullary nailing (EN) alone vs. elastic intramedullary nailing combined with Kirschner wire fixation (EN + K) in the treatment of pediatric radial neck fractures.

**Methods:**

A retrospective cohort study was conducted including 52 pediatric patients with radial neck fractures treated between September 2013 and December 2024 at Wuhan Fourth Hospital. Patients were divided into two groups according to the surgical technique: EN group (*n* = 16) and EN + K group (*n* = 36). Baseline characteristics, perioperative parameters, fracture union time, follow-up duration, complications, and functional outcomes were collected and analyzed. Functional outcomes were evaluated at the final follow-up using Mayo Elbow Performance Score–based grading. Continuous variables were compared using the independent-samples *t*-test, and categorical variables were analyzed using the chi-square test.

**Results:**

There were no significant differences between the two groups in baseline characteristics, including age, sex, time from injury to surgery, Judet classification, or fracture angulation (*P* > 0.05). The EN + K group had a significantly longer operation time (*P* < 0.001) but a shorter immobilization period (*P* < 0.001) and significantly shorter fracture union time compared with the EN group (10.22 ± 2.91 vs. 13.13 ± 2.53 weeks, *P* = 0.001). No postoperative complications were observed in either group. At the final follow-up, the proportion of patients achieving excellent or good functional outcomes was significantly higher in the EN + K group than in the EN group (86.1% vs. 50.0%, *P* = 0.012).

**Conclusion:**

Elastic intramedullary nailing combined with Kirschner wire fixation provides superior fracture stability, promotes faster fracture union, and leads to better functional outcomes compared with elastic intramedullary nailing alone in pediatric radial neck fractures. Despite a longer operation time, the combined technique appears to be a safe and effective treatment option for this patient population.

## 1 Introduction

Pediatric radial neck fractures account for approximately 5%–10% of elbow fractures in children and may result in substantial functional impairment if inadequately treated (1–4). Although relatively uncommon, these injuries are associated with a considerable risk of complications, particularly in cases with significant displacement or instability (5–7).

Treatment strategies for pediatric radial neck fractures depend largely on the degree of fracture displacement and angulation. Minimally displaced fractures are generally managed conservatively, whereas fractures with significant angulation or translation typically require surgical reduction and internal fixation to restore anatomy and function (8–11). Among the available surgical options, elastic intramedullary nailing (EN) and Kirschner wire (K-wire) fixation are commonly used techniques that provide stable fixation for displaced fractures of the radial neck and radial head (8, 11–13).

Elastic intramedullary nailing offers the advantages of minimal invasiveness and internal stabilization while preserving surrounding soft tissues. However, in unstable or severely displaced fractures, EN alone may provide insufficient rotational or angular stability, potentially resulting in loss of reduction or delayed healing. To enhance fracture stability, supplemental K-wire fixation is sometimes employed in combination with EN, although the clinical benefit of this combined strategy remains controversial (12, 14).

Therefore, the purpose of the present study was to compare the clinical outcomes, fracture healing, and functional recovery of pediatric patients with radial neck fractures treated with elastic intramedullary nailing alone vs. elastic intramedullary nailing combined with Kirschner wire fixation.

## 2 Materials and methods

### 2.1 Study design and patient selection

This study was approved by the Wuhan Fourth Hospital Ethical Committee. This retrospective cohort study was conducted at Wuhan Fourth Hospital. Pediatric patients diagnosed with radial neck fractures and treated surgically between September 2013 and December 2024 were consecutively reviewed. The study protocol was approved by the institutional ethics committee, and the requirement for informed consent was waived due to the retrospective nature of the study.

Inclusion criteria were as follows: (1) patients younger than 16 years at the time of injury; (2) radiographically confirmed radial neck fracture; (3) surgical treatment with elastic intramedullary nailing (EN) alone or elastic intramedullary nailing combined with Kirschner wire fixation (EN + K); and (4) a minimum follow-up duration of 6 months. Exclusion criteria included open fractures, pathological fractures, previous ipsilateral elbow injury, and incomplete clinical or radiographic data.

### 2.2 Grouping and treatment allocation

Patients were divided into two groups according to the surgical fixation strategy used. The EN group consisted of patients treated with elastic intramedullary nailing alone, whereas the EN + K group included patients who underwent elastic intramedullary nailing supplemented with percutaneous Kirschner wire fixation.

Fracture stability was the primary determinant of the fixation strategy. Stability is critically important in the treatment of pediatric radial neck fractures, as failure to restore and maintain stability may result in secondary lateral displacement or recurrent angulation even after satisfactory reduction. Fractures were considered unstable when they exhibited specific characteristics, including: (1) Salter–Harris type II fractures with a relatively large metaphyseal fragment, (2) oblique metaphyseal fracture patterns, and (3) impacted or comminuted fractures.

In addition to preoperative radiographic characteristics, intraoperative fracture stability was dynamically assessed under fluoroscopic guidance following reduction and insertion of the elastic intramedullary nail. Stability evaluation included maintenance of fracture alignment during passive elbow flexion–extension and forearm pronation–supination, absence of residual angulation or lateral translation, and resistance to gentle varus–valgus and rotational stress. Fractures demonstrating loss of reduction, persistent displacement, or mechanical instability during dynamic testing were classified as unstable.

When instability was identified intraoperatively, three key issues were addressed simultaneously: correction of lateral displacement, restoration of angulation, and maintenance of mechanical stability. Therefore, supplemental percutaneous Kirschner wire fixation was added to enhance angular and rotational stability and to prevent loss of reduction (EN + K group). In contrast, fractures deemed stable after reduction were treated with EN fixation alone.

### 2.3 Surgical technique

All procedures were performed under general anesthesia with the patient in the supine position. Closed reduction was attempted in all cases under fluoroscopic guidance. For EN fixation, a titanium elastic nail was inserted retrogradely through a distal radial metaphyseal entry point and advanced proximally to achieve reduction and stabilization of the fracture.

In cases where fracture stability was deemed insufficient following EN fixation alone—particularly when residual rotational instability or a tendency toward redisplacement was observed under fluoroscopic assessment—additional percutaneous Kirschner wire fixation was applied to enhance angular and rotational stability.

Rotational instability was specifically assessed intraoperatively by gently rotating the forearm through full pronation and supination under fluoroscopic visualization. Any observable angular change, translation, or redisplacement of the fracture during rotational movement was considered indicative of rotational instability and prompted supplemental K-wire fixation.

All fixation procedures were confirmed fluoroscopically to ensure satisfactory fracture alignment and implant positioning.

### 2.4 Postoperative management

Postoperatively, all patients underwent immobilization using a long-arm cast or splint with the elbow flexed at approximately 90°. The duration of immobilization was determined based on fracture stability and radiographic findings.

Cast removal was performed when early radiographic evidence of callus formation was observed, defined as visible bridging callus across at least two cortices with maintained alignment, together with absence of local tenderness at the fracture site on clinical examination.

After cast removal, patients were encouraged to begin gradual active range-of-motion exercises.

Routine formal physiotherapy was not prescribed for all patients. Active mobilization was initiated immediately after cast removal once radiographic stability was confirmed. Supervised physiotherapy was recommended only in cases with persistent stiffness, delayed recovery of elbow motion, or limited functional improvement after initial home-based exercises.

### 2.5 Data collection

Clinical data collected included age, sex, injured side, time from injury to surgery, Judet fracture classification, initial fracture angulation, operative time, length of hospital stay, duration of immobilization, fracture union time, follow-up duration, and postoperative complications.

Radiographic evaluation was performed using standard anteroposterior and lateral elbow radiographs obtained immediately postoperatively and at follow-up visits. Fracture union was defined as the presence of bridging callus across at least three cortices on anteroposterior and lateral radiographs, disappearance of the fracture line, and absence of local tenderness or pain at the fracture site on clinical examination. Delayed union was defined as insufficient radiographic healing beyond the expected timeframe for pediatric fractures, and nonunion was defined as persistent fracture line visibility without progressive callus formation over at least 6 months of follow-up.

### 2.6 Functional outcome assessment

Functional outcomes were assessed at the final follow-up using a Mayo Elbow Performance Score–based grading system, categorizing outcomes as excellent, good, fair, or poor according to pain, range of motion, stability, and functional capacity. All assessments were performed by attending orthopedic surgeons who were not involved in the surgical procedures.

### 2.7 Statistical analysis

Statistical analyses were performed using SPSS software (version 23.0; IBM Corp., Armonk, NY, USA). Continuous variables were presented as mean ± standard deviation and compared using the independent-samples *t*-test or Mann–Whitney *U*-test, as appropriate. Categorical variables were expressed as frequencies and percentages and analyzed using the chi-square test or Fisher's exact test. A *P* value < 0.05 was considered statistically significant.

## 3 Results

### 3.1 Patient characteristics

A total of 52 pediatric patients with radial neck fractures were included in this retrospective cohort study. According to the surgical technique applied, 16 patients were assigned to the EN group and 36 patients to the EN + K group. Baseline demographic and clinical characteristics are summarized in Table 1. There were no significant differences between the two groups with respect to age, sex distribution, time from injury to surgery, Judet fracture classification, or initial fracture angulation (all *P* > 0.05), indicating good comparability between the groups.

**Table 1:** Clinical characteristics of the whole cohort.

Variables	Total (*n* = 52)	EN group	EN + K group	*P*
Average age (years), mean ± SD	8.33 ± 2.83	7.94 ± 2.96	8.50 ± 2.80	0.514
Gender				0.515
Male	23 (44.2)	6 (37.5)	17 (47.2)	
Female	29 (55.8)	10 (62.5)	19 (52.8)	
Time from injury to surgery (h)	83.63 ± 30.98	93.50 ± 31.26	79.25 ± 30.26	0.127
Judet classification				1.000
II	2 (3.8)	1 (6.3)	1 (2.8)	
III	34 (65.4)	10 (62.5)	24 (66.7)	
IV	16 (30.8)	5 (31.3)	11 (30.6)	
Fracture angulation (°)	46.17 ± 16.28	44.96 ± 13.97	46.70 ± 17.36	0.726

### 3.2 Perioperative and clinical outcomes

Comparisons of perioperative parameters and clinical outcomes are presented in Table 2. The mean operation time was significantly longer in the EN + K group than in the EN group (61.03 ± 26.17 vs. 26.25 ± 4.28 min, *P* < 0.001). However, patients treated with EN + K required a significantly shorter period of postoperative immobilization compared with those treated with EN alone (4.17 ± 0.38 vs. 4.88 ± 0.34 weeks, *P* < 0.001). There was no significant difference in hospital stay between the two groups (*P* = 0.221).

**Table 2:** Comparison of clinical outcomes between the two groups.

Variables	Total (*n* = 52)	EN group	EN + K group	*P*
Operation time (min)	50.33 ± 27.17	26.25 ± 4.28	61.03 ± 26.17	<0.001
Immobilization time (weeks)	4.38 ± 0.49	4.88 ± 0.34	4.17 ± 0.38	<0.001
Hospital stay (days)	16.21 ± 2.78	15.50 ± 4.50	16.53 ± 1.48	0.221
Fracture union time (weeks)	11.12 ± 3.09	13.13 ± 2.53	10.22 ± 2.91	0.001
Follow-up duration (months)	8.79 ± 2.10	8.69 ± 2.09	8.83 ± 2.13	0.820
Complications, *n* (%)	0	0	0	—

Fracture union was achieved in all patients. The mean fracture union time was significantly shorter in the EN + K group compared with the EN group (10.22 ± 2.91 vs. 13.13 ± 2.53 weeks, *P* = 0.001). The mean duration of follow-up did not differ significantly between the two groups (8.69 ± 2.09 vs. 8.83 ± 2.13 months, *P* = 0.820). No postoperative complications, including infection, nonunion, or neurovascular injury, were observed in either group.

To further assess the clinical relevance of the observed differences, standardized effect sizes (Cohen's d) were calculated (Table 3). The magnitude of between-group differences was large for fracture union time, and very large for operation time and immobilization time, indicating substantial clinical effects in addition to statistical significance.

**Table 3:** Effect size analysis (Cohen's d) for between-group comparisons.

Variables	Cohen's d	Interpretation
Operation time (min)	1.58	Very large
Immobilization time (weeks)	1.93	Very large
Fracture union time (weeks)	1.04	Large

### 3.3 Functional outcomes

Functional outcomes assessed at the final follow-up using the Mayo Elbow Performance Score–based grading are shown in Table 4. In the EN group, outcomes were rated as excellent in 2 patient (12.5%), good in 6 patients (37.5%), fair in 7 patients (43.8%), and poor in 1 patient (6.3%). In contrast, the EN + K group demonstrated excellent outcomes in 19 patients (52.8%), good outcomes in 12 patients (33.3%), and fair outcomes in 5 patients (13.9%), with no poor outcomes recorded.

**Table 4:** Subgroup analysis of functional outcomes (mayo elbow performance score–based grading).

Variables	Total (*n* = 52)	EN group	EN + K group	*P*
Excellent, *n* (%)	21 (40.4)	2 (12.5)	19 (52.8)	0.006
Good, *n* (%)	18 (34.6)	6 (37.5)	12 (33.3)	
Fair, *n* (%)	12 (23.1)	7 (43.8)	5 (13.9)	
Poor, *n* (%)	1 (1.9)	1 (6.3)	0 (0.0)	
Excellent + Good, *n* (%)	39 (75.0)	8 (50.0)	31 (86.1)	0.012

Overall, the proportion of patients achieving excellent or good functional outcomes was significantly higher in the EN + K group compared with the EN group (86.1% vs. 50.0%, *P* = 0.012).

### 3.4 Representative case

**Figure 1:**
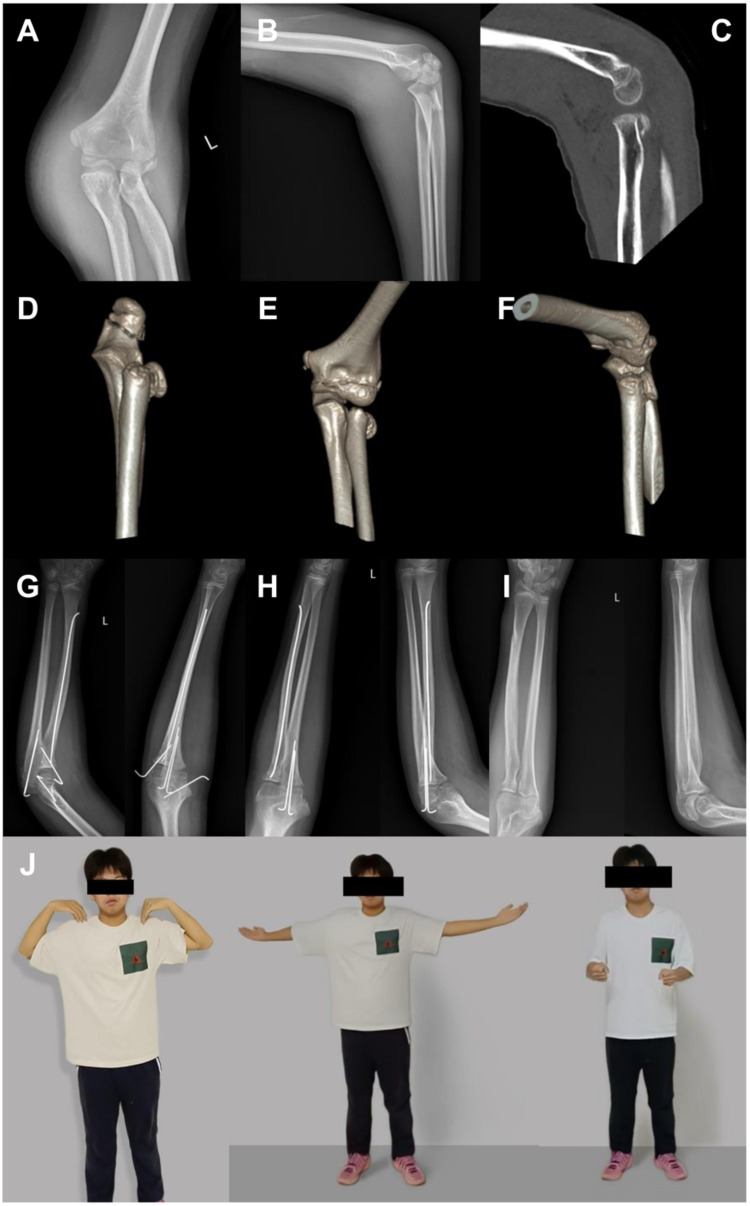
Representative case of an unstable pediatric radial neck fracture treated with elastic intramedullary nailing combined with Kirschner wire fixation. **(A,B)** Preoperative anteroposterior and lateral radiographs showing a severely displaced radial neck fracture. **(C–F)** Preoperative computed tomography images demonstrating marked fracture displacement and collapse of the radial neck, associated with olecranon and medial epicondyle fractures. **(G)** Radiograph at 1 month postoperatively showing maintained reduction and early callus formation. **(H)** Radiograph at 2 months postoperatively demonstrating progressive fracture healing. **(I)** Radiograph at 8 months postoperatively confirming complete fracture union. **(J)** Clinical photograph at final follow-up showing full elbow flexion, extension, and forearm rotation.

A representative case is presented in Figure 1.

## 4 Discussion

The present study demonstrates that elastic intramedullary nailing combined with supplemental Kirschner wire fixation results in faster fracture union and improved functional outcomes compared with elastic intramedullary nailing alone in pediatric radial neck fractures. Although all fractures ultimately achieved bone union, the time to radiographic stabilization was significantly shorter in the EN + K group, suggesting a clinically meaningful advantage of enhanced fixation stability in the early phase of fracture healing.

Early fracture union is particularly important in pediatric elbow injuries, as prolonged immobilization is closely associated with elbow stiffness and restricted forearm rotation (15–18). In our cohort, a significantly higher proportion of patients in the EN + K group achieved earlier radiographic union, which translated into shorter immobilization duration and superior functional recovery at final follow-up. This finding is consistent with previous studies reporting that fracture stability is a key determinant of healing speed and functional outcome in pediatric radial neck fractures (12, 19, 20). Although elastic intramedullary nailing provides internal support with minimal soft-tissue disruption, EN alone may be insufficient to control rotational and angular instability in severely displaced fractures, potentially delaying consolidation.

The biomechanical rationale for combining EN with K-wire fixation lies in the complementary stabilization mechanisms of the two implants. Elastic intramedullary nails primarily provide axial and bending stability but offer limited resistance to rotational forces, particularly in fractures with comminution or marked angulation (21, 22). Supplemental K-wire fixation can improve rotational control and maintain reduction, thereby minimizing micromotion at the fracture site and facilitating callus formation. Similar principles have been emphasized in prior biomechanical and clinical studies addressing unstable pediatric elbow fractures (23–25).

Functional outcomes in the present study suggest a potential advantage of combined fixation in selected unstable fracture patterns rather than supporting its routine use in all cases. A significantly greater proportion of patients in the EN + K group achieved excellent or good functional results compared with those treated with EN alone. However, this finding should be interpreted in the context of fracture stability, as supplemental K-wire fixation was primarily applied in fractures demonstrating intraoperative instability. Stable fixation allowing earlier mobilization is a well-recognized factor in preventing postoperative elbow stiffness, one of the most common long-term complications following proximal radial injuries (18, 26). In contrast, concerns regarding potential over-fixation or additional soft tissue irritation with K-wires were not substantiated in our series, as no increase in complications was observed.

Importantly, the addition of K-wire fixation did not result in a higher complication rate. No cases of pin-tract infection, neurovascular injury, nonunion, or implant-related complications were identified in either group. Previous literature has reported complication rates ranging from 10% to 40% for surgically treated pediatric radial neck fractures, depending on fracture severity and surgical technique (2). The absence of complications in our study may be attributed to meticulous surgical technique, strict adherence to indications for supplemental fixation, and timely removal of K-wires once radiographic healing was evident.

Although fracture union occurred earlier in the EN + K group, long-term bone healing was ultimately achieved in all patients, regardless of fixation method. This finding aligns with previous studies indicating that pediatric fractures generally have a high healing potential due to robust biological remodeling capacity (12). Therefore, both EN alone and EN combined with K-wire fixation can be considered effective surgical options. However, our results suggest that EN + K fixation may be preferable in unstable fracture patterns where achieving and maintaining reduction is challenging.

Several factors known to influence outcomes in pediatric radial neck fractures, including age, fracture displacement, and associated injuries, were evenly distributed between groups in this study (20). Notably, neither Judet classification nor the presence of associated injuries was associated with delayed healing or inferior outcomes, which may reflect the effectiveness of stable internal fixation and standardized postoperative management.

The present study has several strengths. It includes a well-defined cohort with comparable baseline characteristics, standardized surgical techniques, and consistent follow-up protocols. Functional outcomes were systematically assessed, and radiographic union was evaluated using predefined criteria. However, some limitations should be acknowledged. First, the retrospective design may introduce inherent selection bias. However, the choice of surgical technique was primarily determined by fracture stability rather than surgeon preference. Stable fractures were typically treated with elastic intramedullary nailing alone, whereas unstable fractures required supplemental Kirschner wire fixation to restore and maintain fracture stability. In our study, fractures with certain characteristics were considered unstable, including Salter–Harris type II fractures with a relatively large metaphyseal fragment, oblique metaphyseal fracture patterns, and impacted or comminuted fractures. Second, the sample size—particularly in the EN group—was relatively small, limiting the power for subgroup analyses. Furthermore, although fracture severity (e.g., Judet classification and initial angulation) was recorded, the relatively small cohort size precluded robust stratified analyses to determine whether specific fracture subtypes derived greater benefit from supplemental K-wire fixation. Additional subgroup analyses would have substantially reduced statistical power and increased the risk of type II error. Therefore, larger prospective studies are needed to better clarify the interaction between fracture displacement, stability patterns, and treatment effectiveness. Third, fracture union was assessed at predefined follow-up intervals rather than as a continuous variable, which may have reduced the precision of healing time comparisons. Finally, long-term outcomes beyond skeletal maturity were not evaluated. The mean follow-up duration of approximately 8–9 months may be insufficient to fully assess long-term functional recovery, recurrence of complications, or potential growth-related sequelae in pediatric patients. Although no growth disturbances or late complications were observed during the available follow-up period, proximal radial fractures involve the metaphyseal–physeal region and may theoretically affect future elbow alignment and forearm rotation. Therefore, longer-term follow-up extending beyond one year, ideally until skeletal maturity, is warranted to confirm the durability and safety of the combined fixation strategy. Future prospective, multicenter studies with larger sample sizes and standardized functional assessments are needed to further clarify the role of combined fixation strategies and to identify fracture patterns that benefit most from supplemental K-wire stabilization.

## 5 Conclusion

Elastic intramedullary nailing combined with Kirschner wire fixation provides greater fracture stability, accelerates fracture union, and leads to superior functional outcomes compared with elastic intramedullary nailing alone in pediatric radial neck fractures. Although the combined technique is associated with a longer operative time, it does not increase complication rates and appears to be a safe and effective treatment option, particularly for unstable or severely displaced fractures.

## Data Availability

The raw data supporting the conclusions of this article will be made available by the authors, without undue reservation.
